# *Brassica Rapa SR45a* Regulates Drought Tolerance via the Alternative Splicing of Target Genes

**DOI:** 10.3390/genes11020182

**Published:** 2020-02-10

**Authors:** Muthusamy Muthusamy, Eun Kyung Yoon, Jin A Kim, Mi-Jeong Jeong, Soo In Lee

**Affiliations:** 1 Department of Agricultural Biotechnology, National Institute of Agricultural Sciences (NAS), RDA, Jeonju 54874, Korea; biotech.muthu@gmail.com (M.M.); jakim72@korea.kr (J.A.K.); center1097@korea.kr (M.-J.J.); 2 Department of Chemical and Biomolecular Engineering, National University of Singapore, 4 Engineering Drive 4, Singapore 117585, Singapore; cheyek@nus.edu.sg

**Keywords:** serine/arginine-rich proteins, drought tolerance, alternative splicing, BrSR-like 3, thermotolerance

## Abstract

The emerging evidence has shown that plant serine/arginine-rich (SR) proteins play a crucial role in abiotic stress responses by regulating the alternative splicing (AS) of key genes. Recently, we have shown that drought stress enhances the expression of *SR45a* (also known as *SR-like 3*) in *Brassica rapa.* Herein, we unraveled the hitherto unknown functions of *BrSR45a* in drought stress response by comparing the phenotypes, chlorophyll a fluorescence and splicing patterns of the drought-responsive genes of Arabidopsis *BrSR45a* overexpressors (OEs), homozygous mutants (SALK_052345), and controls (Col-0). Overexpression and loss of function did not result in aberrant phenotypes; however, the overexpression of *BrSR45a* was positively correlated with drought tolerance and the stress recovery rate in an expression-dependent manner. Moreover, OEs showed a higher drought tolerance index during seed germination (38.16%) than the control lines. Additionally, the overexpression of *BrSR45a* induced the expression of the drought stress-inducible genes *RD29A, NCED3*, and *DREB2A* under normal conditions. To further illustrate the molecular linkages between *BrSR45a* and drought tolerance, we investigated the AS patterns of key drought-tolerance and *BrSR45a* interacting genes in OEs, mutants, and controls under both normal and drought conditions. The splicing patterns of *DCP5, RD29A, GOLS1, AKR, U2AF,* and *SDR* were different between overexpressors and mutants under normal conditions. Furthermore, drought stress altered the splicing patterns of *NCED2, SQE, UPF1*, *U4/U6-U5 tri-snRNP-associated protein*, and *UPF1* between OEs and mutants, indicating that both overexpression and loss of function differently influenced the splicing patterns of target genes. This study revealed that *BrSR45a* regulates the drought stress response via the alternative splicing of target genes in a concentration-dependent manner.

## 1. Introduction

Plants respond to abiotic and biotic stresses and developmental stimuli by transcriptional reprogramming through constitutive and alternative RNA splicing events. RNA splicing is a highly ordered and dynamic posttranscriptional modification [1] catalyzed by a highly conserved ribonucleoprotein complex known as the spliceosome comprising five small nuclear ribonucleoproteins (snRNPs) (U1, U2, U4/U6, and U5) and numerous non-snRNP proteins, including serine/arginine-rich (SR) proteins [2,3]. The SR proteins have a modular structure (20 to 75 kDa) consisting of one or two N-terminal RNA recognition motifs (RRMs) and serine and arginine-rich C-terminal domains (RS domains with multiple RS dipeptide repeats) [4] that can bind and interact with target pre-mRNAs and proteins to modulate splicing [5]. The SR protein and pre-mRNA interactions either activate or repress constitutive and alternative splicing for the maintenance of cellular and tissue homeostasis [6,7]. The phosphorylation and/or dephosphorylation of the RS domain alters the binding affinity of SR proteins for pre-mRNAs and other proteins, thus affecting splicing differently [8]. Protein kinases phosphorylate SR proteins, which initiate spliceosome complex formation [3]. The spliceosome performs the two transesterification reactions that are necessary to excise introns and join together the selected exons [1]. In response to stress, plant SR genes initially undergo alternative splicing prior to the alternative splicing of the target genes, including stress response genes [6]. The stress-dependent alternative splicing of SR proteins, which are key splice factors for protein-encoding genes, have distinct biological functions [9,10], suggesting the existence of a posttranscriptional level regulatory mechanism for stress response genes [11].

The nomenclature of SR proteins has been designed solely according to their sequence properties; they comprise one or two N-terminal RRMs (RBDs; PF00076) followed by a downstream RS domain of at least 50 amino acids with 40% RS content characterized by consecutive RS or SR repeats [12]. Nonetheless, two RS domains at the N- and the C-termini are observed in *atSR45a* [13]. Moreover, some AS events in *A. thaliana* genes and in three Brassica species (*B. rapa, B. oleracea*, and *B. napus*) have been evolutionarily conserved [14]. The model plant *Arabidopsis thaliana* encodes 18 SR proteins, while *Brassica rapa* has 25 SR genes (BrSR) that are classified into six subfamilies, named SR, RSZ, SC, SCL, RS2Z, and RS according to their distinct domain organization; most of them are responsive to oxidative, cold, and heat stress [15]. The SR genes are known to be actively alternatively spliced under stress conditions [16]. Heat and drought stress induce the alternative splicing of *atSR45a* (AT1G07350.1 and AT1G07350.2) and enhance its expression [17]. The levels of the transcripts *atSR45a, atSR30*, and *SF2/ASF-like SR* were increased by high-light irradiation and salinity stress [13]. Nonetheless, the determination of the *SR45a* target genes and their splicing patterns under particular stress conditions will provide vital clues as to their functional role in stress responses, which have yet to be identified. SR proteins participate in multiple signaling pathways, including the glucose signaling pathway [18], the nonsense-mediated mRNA decay pathway (NMD), which is involved in the RNA surveillance system [19], the ABA signaling pathway [20], the autonomous flowering pathway [21], and stress response gene expression pathways. There is mounting evidence implicating *AtSR* genes in the signal transduction of the abscisic acid (ABA) phytohormone, a key modulator of various abiotic stress responses (drought, high salinity, light, cold, and heat) in plants [5,22]. In addition, SR-mediated pre-mRNA splicing processes were also influenced in a concentration-dependent manner [16]. As central coordinators of plant abiotic stress responses, SR genes have drawn the attention of plant researchers focusing on the development of stress-resilient crops. Recently, a high-throughput approach was implemented to profile the binding targets of SR proteins, which are crucial stress response splicing factors in plants [19]. For example, *AtSR45* either directly or indirectly interacts with 4000 RNAs/genes, which mostly include abscisic acid (ABA) signaling genes [23]. Herein, we attempted to characterize the functions of the *BrSR45a* gene (also known as *BrSR-like 3*) in drought and heat stress responses by gathering phenotypic and molecular evidence supporting our hypothesis from *BrSR45a* overexpressors and mutant and control Arabidopsis lines. This study will increase our understanding of the molecular underpinnings of the relationship between *BrSR45a* and drought stress tolerance.

## 2. Materials and Methods 

### 2.1. Promoter Isolation, Sequencing, and Genetic Transformation for the Overexpression of the BrSR45a Promoter:GUS

The genomic DNA encoding the promoter region of Bra024988 (A06:23484367.23486104) was retrieved from the *B. rapa* genome sequences available in the BRAD database (version 1.5; accessed on 17.12.2015). The promoter (2 kb) region (Supplementary Figure S1) was amplified with specific primers flanked with *Eco*RV and *Spe1* restriction enzyme sites (forward: 5‘-ggGATATCGACCAAGATAAAGTTTGCATCA-3‘; reverse: 5′ggACTAGTTGCTGAGAAACTCTTAGAAATC-3′) and ligated into a pGEM-T Easy vector, which was transformed into DH-5α cells for sequence confirmation and then cloned into an expression vector (pCAMBIA1391Z) at the *Eco*RI site according to the method described previously [24]. The generated binary vector (pCAMBIA1391Z + *BrSR45a*:*GUS*) was genetically transformed with the Agrobacterium strain (GV3101) into *A. thaliana*. The seeds from *BrSR45a*:*GUS* transgenic Arabidopsis lines were surface-sterilized with 70% ethanol for 15 min and with 100% ethanol for 2 min prior to plating on Murashige and Skoog (MS) medium supplemented with vitamins, 1% sucrose, and 0.25% Phytagel (Sigma-Aldrich, St Louis, MO) for the GUS staining assay. To induce synchronous germination, the seeds were stratified at 4 °C for 3 d in the dark and then transferred to a growth chamber. To identify the histochemical localization of GUS activity resulting from *BrSR45a* promoter activity, whole plants and leaf samples were directly used for the GUS assay with the β-Glucuronidase Reporter Gene Staining Kit (Sigma-Aldrich, St. Louis, MO, USA) according to the manufacturer’s instructions. To identify the putative cis-acting elements of *BrSR-45a*, the promoter region (−2000 bp to +1 bp) was searched against known motifs in the plantCARE (http://bioinformatics.psb.ugent.be/webtools/plantcare/html/) database. All tools and programs mentioned in this study were used with the default settings unless otherwise specified.

### 2.2. Plant Materials, RNA Extraction, and Isolation of BrSR45a

Sterilized seeds from *B. rapa* cv. Dongbu was allowed to pre-germinate in moistened Whatman^TM^ (3 MM) filter paper in Petri dishes at 22 °C for 3 d. Subsequently, the uniformly sized seedlings were used as replicates (*n* = 3) and were transferred to bed soil, and the leaf tissues were collected after 3 weeks in greenhouse conditions. The leaf tissues were immediately frozen in liquid nitrogen and stored at −80 °C for subsequent RNA and genomic DNA preparation. Total RNA was isolated from 100 mg of powdered sample using the RNeasy Mini kit (QIAGEN, Germany). To eliminate DNA contamination, the total RNA was treated with DNase I (QIAGEN, Germany) at 28 °C for 15 min. The purified total RNA (3 μg) was used as a template for first-strand cDNA synthesis using amfiRivert cDNA Synthesis Platinum Master mix according to the manufacturer′s protocols. From the 1:10 diluted cDNA, the *BrSR45a* coding region (1050 bp) was amplified by polymerase chain reaction (PCR) with *att*B-flanking, gene-specific forward (5′-AAAAAGCAGGCTATGTCTTACTCAAGATACTCTC-3′), and reverse primers (5′-AGAAAGCTGGGTTTATGGGCTGACTGATCGAG-3′). The PCR conditions were as follows: 95 °C for 3 min followed by 35 cycles of 95 °C for 10 s, 55 °C for 30 s, and 72 °C for 2 min, with a final extension at 72 °C for 10 min. For hormone, drought and heat stress treatments, 7-d-old seedlings were exposed to 100 μM ABA, 250 mM D-mannitol or no irrigation conditions and high temperature (37 °C) at growth chambers, respectively. The whole seedlings were used for *BrSR45a* expression analysis in this study.

### 2.3. Vector Construction, Genetic Transformation, and Selection of Homozygous AtSR45a Mutants

To create the Gateway Entry clone, the PCR products were cloned by a BP reaction into pDONR221, which was later recombined with the destination vector pH2GW7 by an LR reaction prior to Agrobacterium (*A. tumefaciens* strain, GV3101)-mediated genetic transformation into Arabidopsis (Col-0) using the floral dip method. For genetic transformation, *A. thaliana* Columbia (Col-0) seeds were surface-sterilized with 70% ethanol for 15 min and 100% ethanol for 2 min and plated on Murashige and Skoog (MS) medium supplemented with vitamins, 1.5% sucrose, and 0.25% phytagel (Sigma, St Louis, MO). The plates were stratified at 4 °C in the dark for 2 days to induce synchronous germination and transferred to a growth chamber (16-h light/8-h dark photoperiod at 23 °C) for transformation. Several hygromycin-resistant homozygous transgenic lines were selected and used for gene expression analyses, drought phenotyping, and other studies. The overexpression of *BrSR45a* in transgenic Arabidopsis was confirmed by qRT-PCR analysis with the CFX96TM Real-Time PCR Detection System (Bio-Rad, California, USA). A set of primers (FP-5′-AAGCAAGTCACGGAGCGTAT-3′; RP-5′TTATGGGCTGACTGATCGA-3′) along with AccuPower^®^2X GreenStar Master Mix (Bioneer, Korea) was used for the PCR reaction at 95 °C for 5 min followed by 40 cycles of 95 °C for 15 s and 60 °C for 30 s for the relative quantification of *BrSR45a*. The expression was normalized to that of *AtActin2* using specific primers (FP-5′-TCGGTGGTTCCATTCTTGCT-3′; RP-5′-GCTTTTTAAGCCTTTGATCTTGAGAG-3′). Similarly, the expression of *AtDREB2A, AtNCED3, AtKIN1*, and *AtRD29A* in the transgenic lines were measured in this study. Each treatment has three biological and technical replicates. The expression levels were calculated as the mean signal intensity across three replicates, and the standard error was presented as error bars in graphs. The homozygous *AtSR45a* mutant was isolated from the seedlings of SALK_052345 using PCR-based genotyping methods as recommended by ABRC (Supplementary Figure S2).

### 2.4. Stress Tolerance Assay

The uniform size seedlings were transferred and grown in soil with 45–50% volumetric water content for 3 weeks. To impose drought stress, watering was withheld for 17 days consecutively, and the chlorophyll a fluorescence transient curves (OJIP curves) were determined for 15 days after drought stress in dark-adapted leaves using FluorPen FP 110 (Photon System Instruments) (Supplementary Figure S3). The leaf length and width were also measured. The selected transgenic lines (T1) harboring 35S:*BrSR45a* were treated under in vitro conditions to study drought and heat stress tolerance. To test the basal drought tolerance through seed germination, sterilized seeds (50–250 seeds for each replicate) of OE and Col-0 were sown on 3MM filter papers moistened with different percentages of PEG6000 solution (10%, 15%, and 20%), while the controls were maintained in water. The germination percentage (G%) was calculated after 7 d of incubation at 23 °C in 60% relative humidity with a 16 h photoperiod in a growth chamber. The drought tolerance index ($DTI=\frac{G\%\;under\;droughtstress}{G\%\;under\;control}\;X\;100$) of each line was calculated as described previously [25]. Similarly, the heat stress responses of 9-day-old seedlings of OEs, mutants, and controls grown on plates were tested by immersing in a water bath for 35 min at 45 °C. After an 8-d recovery period, the heat stress tolerance was estimated as percentage of seedlings survival under stress conditions.

### 2.5. Analysis of Alternative Splicing

To gain insight into the alternative splicing pattern of drought-tolerant genes in *BrSR45a* overexpressors, mutants and Col-0, the semi-quantitative qRT-PCR analysis of the leaf transcriptomes of all seedlings before and after drought stress was performed. For this purpose, primers were designed (Supplementary Table S1) for the amplification of splice variants using the NCBI Primer-BLAST tool, and the respective target sequences were retrieved from the NCBI gene database. The *BrSR45a* protein interaction network was predicted using the string-db database at http://string-db.org (Supplementary Figure S4). The RNA extraction and first-strand cDNA synthesis were performed as described above. The amplicons were resolved using a 2.5% agarose gel by electrophoresis and visualized with Inclone^TM^ Safe Gel Stain (Inclone, Korea).

### 2.6. Statistical Analyses

All the treatments mentioned in this study had at least three independent biological and technical replicates. For gene expression analyses, the mean signal intensity across three replicates was considered and the standard error was presented as error bars in graphs. By default, the results were subjected to one-way analysis of variance (one-way ANOVA) and the means were compared by Tukey′s HSD test at http://www.astata.com/. Statistics by ANOVA test are shown; * *P* < 0.05, ** *P* < 0.001, *** *P* < 0.0001, and NS, no significance.

## 3. Results

Herein, we attempted to characterize *BrSR45a* through its heterologous expression in Arabidopsis. We investigated the impact of its overexpression and loss of function on the leaf and root phenotypes, chlorophyll a fluorescence, stress responses to drought and heat stress and, finally, the alternative splicing of genes that have been implicated in drought tolerance and BrSR45a interacting partners. In addition, we also analyzed the spatial expression of *BrSR45a* promoters in transgenic reporter lines.

### 3.1. Abiotic Stress Response Promoter Motifs and GUS Activity in Transgenic Reporter Lines (BrSR45a::GUS)

**Figure 1:**
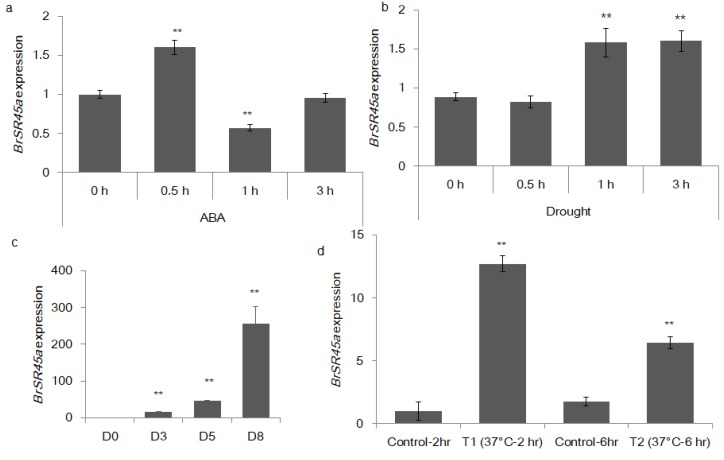
ABA, drought and heat stress affected the expression pattern of the *B. rapa SR45a* gene. The bar chart depicts the relative quantification of transcripts of *BrSR45a* during the exogenous application of ABA (**a**), drought (**b**,**c**) and heat stress (**d**), as measured using a quantitative RT-PCR assay; their expression was normalized to that of the *B. rapa* actin gene (** represents statistical significance). D0, D3, D5, and D8 represent the number of days plants were subjected to drought stress.

The promoter analysis showed that the SR45a promoter sequences had three important cis-acting elements that were responsive to abiotic stress. The promoter motifs were ABRE (GTGCAT), MYB (TAACTG), and HSE (AAAAATTTTC), which are known to participate in plant responses to ABA, drought and heat stress, respectively. Our preliminary analysis revealed that the exogenous application of stress hormones, ABA, heat and drought stress for different time intervals regulated *BrSR45a* expression (Figure 1). 

**Figure 2:**
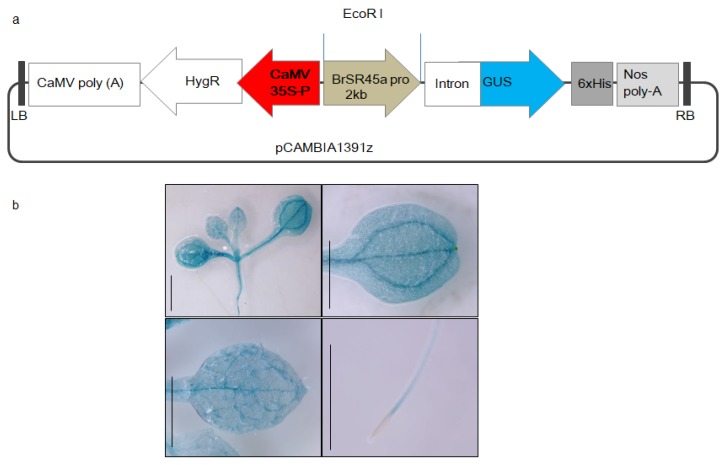
Development of transgenic reporter Arabidopsis lines expressing *GUS* under the promoter activity of *B. rapa SR45a.*The representative vector map of pCAMBIA1391Z (**a**), which was used for *BrSR45a* promoter characterization and (**b**) the measurement of GUS activity in transgenic reporter Arabidopsis lines (*BrSR45a* promoter::*GUS*) expressing *GUS* under the promoter of *BrSR45a.*

The presence of promoter motifs and *SR45a* regulation by ABA and drought stress indicated that the stress responses of *SR45a* might be mediated by its promoter motifs. To understand the spatiotemporal expression of *SR45a*, transgenic Arabidopsis reporter lines expressing GUS via the promoter of *BrSR45a* were developed (Figure 2a). Histochemical localization in transgenic reporter lines revealed that *BrSR45a* expression was prominent in shoots, vascular root tissues, and the primary and secondary veins of developing leaves in young plants (Figure 2b). The presence of GUS activity proved the role of *SR45a* in plant development.

### 3.2. Phylogeny, Phenotypes, and Drought Stress Responses of BrSR45a

**Figure 3:**
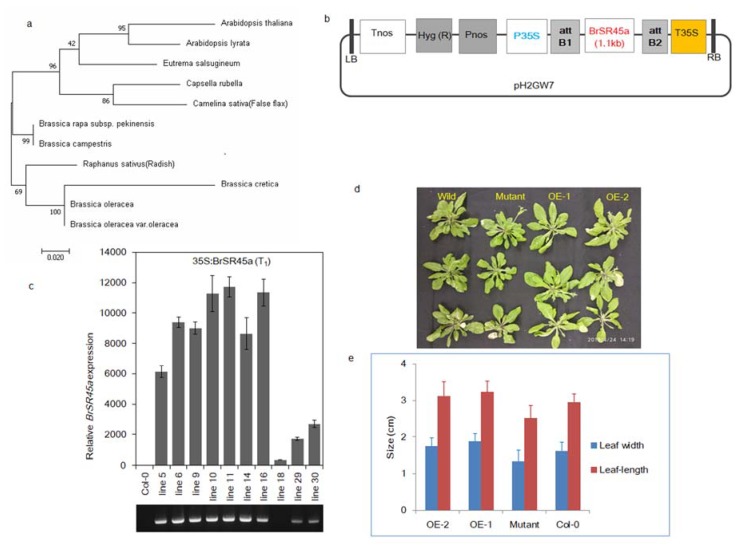
Heterologous expression of *BrSR45a* in Arabidopsis and its corresponding growth parameters. (**a**–**c**) denote the phylogenetic relationships, *BrSR45a* expression cassette, and expression level of *BrSR45a* in the transgenic Arabidopsis lines, respectively, while (**d**,**e**) compares the leaf growth parameters of *BrSR45a* overexpressors with that in *SR45a* knock-out mutants and control lines (Col-0).

The phylogenetic analysis of the BrSR45a (BrSR-like 3) protein sequence (361 AA) in Brassica and Brassicaceae members (Figure 3a) showed a fair amount of conservation in the amino acid residues. Further analysis revealed that *BrSR45a* is more closely related to that in *B. campestris* in Brassica and *Camelina sativa* and *Raphanus sativus* in Brassicaceae. Brassica members, such as *B. oleracea* and *B. cretica,* were placed in different clades, indicating that evolutionary divergence occurred during natural selection among Brassica members. To functionally characterize the *BrSR45a*gene, it was PCR-amplified from *B. rapa cv.* Dongbu and overexpressed in *A. thaliana* (Col-0) using Agrobacterium-mediated genetic transformation (Figure 3b, c), and the resulting plants were compared in terms of their phenotype with SR45a knock-out mutants and the control Col-0. 

**Figure 4:**
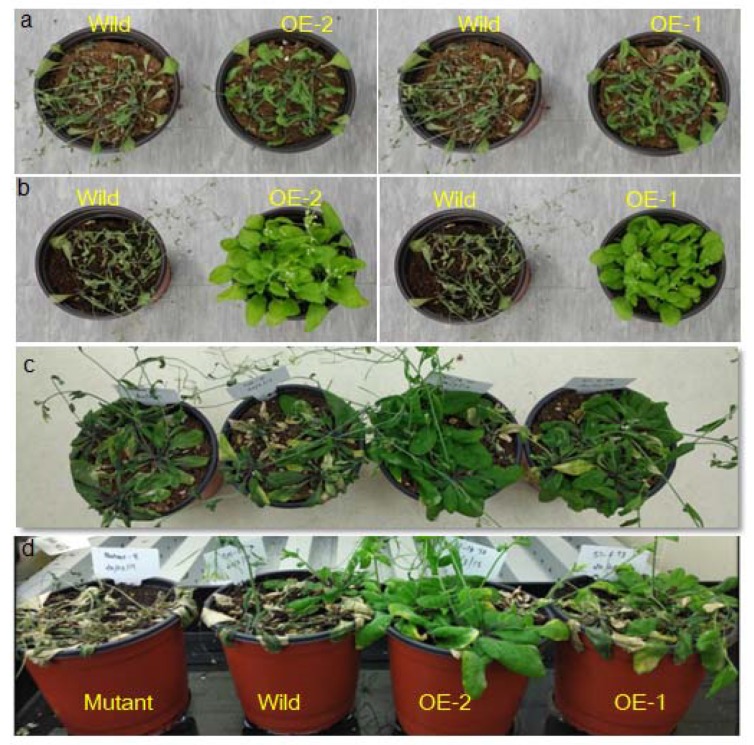
Drought phenotyping of *BrSR45a* overexpressors (OE-1, OE-2), *SR45a* mutants and wild type lines subjected to progressive drought stress. (**a**,**c**) depict plants subjected to 11 and 17 d of progressive drought stress, while (**b**,**d**) represent plants after a stress recovery period of 1 d.

No aberrant phenotypes were observed in them, except that the leaves in the mutant were slightly narrower than those in the *BrSR45a*-overexpressing and Col-0 Arabidopsis lines; however, no significant difference between OE and Col-0 was found for leaf length and leaf width (Figure 3d,e). Similarly, no difference was observed in the root phenotypes among them, although SR45a:*GUS* constructs showed activity in vascular root tissues. However, we developed our main interest in the drought stress responses of OEs and mutants based on preliminary studies of their responses to drought, the stress hormone ABA, and promoter motifs (Figure 1). We exposed OE, mutant and Col-0 lines to progressive drought stress for 17 consecutive days by suspending irrigation. The drought-induced subsequent stress was confirmed through the measurement of molecular markers of drought and oxidative stress. The expression of stress markers (*DREB2A, APX6, MDAR1, DHAR1*, and *GR3*) during drought stress was progressively upregulated, as shown via quantitative PCR assays (Supplementary Figure S5). To measure the drought stress impact on photosystem II in each phenotype, chlorophyll a fluorescence transients (OJIPs) in dark-adopted leaves during progressive drought stress were measured (Supplementary Figure S3). The comparative analysis of the OJIPs showed that the O-J, J-I, and I-P phases in the overexpressors were positive and relatively higher than those in the controls, Col-0 and SR45a mutants during drought stress. Interestingly, all phases of OJIP in mutants were reduced under stress in comparison with that in control plants. Moreover, the comparative drought phenotyping indicated that OEs could tolerate the dehydration imposed by soil moisture deficit stress, whereas mutants and Col-0 succumbed to stress and collapsed completely under stress conditions (Figure 4a–d). Upon rewatering, OE lines with the highest expression (Supplementary Figure S6) showed a better recovery rate than OE lines with moderate expression. Based on these results, we presumed that SR45a might participate in drought stress tolerance and/or stress recovery mechanisms, but its efficiency may depend on the magnitude of the expression level. The preliminary study of *BrSR45a* in *B. rapa* also showed that drought stress induced its expression (Figure 1). Exposure to exogenous ABA also induced the expression of *BrSR45a*, suggesting that *BrSR45a* might play a role in both drought and ABA responses. 

### 3.3. Expression Pattern of Drought and Antioxidative Stress Response Genes

**Figure 5:**
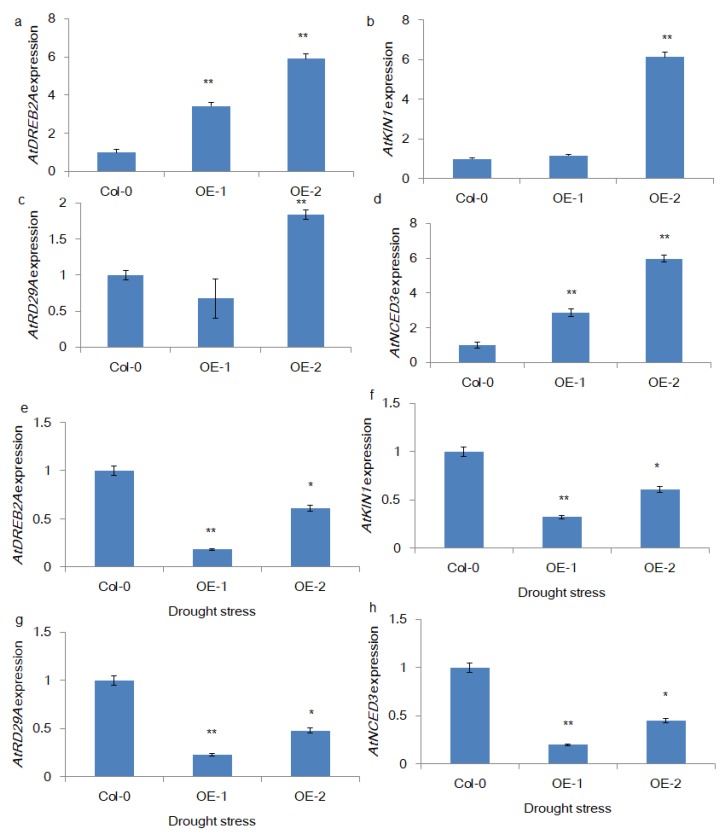
Expression patterns of stress response genes in transgenic Arabidopsis seedlings. The relative quantification of the expression of stress response genes in transgenic Arabidopsis seedlings under normal (**a**–**d**) and drought stress (11-d) conditions (**e**–**h**) was measured using qRT-PCR (**, * represents the statistical significance calculated from triplicate measurements).

The literature shows that stress response gene expression determines drought stress responses, including tolerance and avoidance, in crop plants. Therefore, we attempted to measure the expression of some of the well-known genes in the drought stress response pathway, namely, *AtDREB2A, AtNCED3, AtKIN1*, and *AtRD29A*. The qRT-PCR-based relative quantification results (Figure 5a–d) showed that all 4 genes were upregulated in transgenic lines compared with control plants, indicating that the overexpression of *BrSR45a* might induce stress response gene expression in transgenic plants. Interestingly, their expression in transgenic lines was significantly declined under drought conditions when compared to control lines.

The drought tolerance of plants is partially attributed to their inbuilt antioxidative system, comprising several antioxidants and free radical scavenging enzymes. Hence, we also investigated the expression of antioxidative genes such as *AtAPX1, AtGR1, AtSOD1, AtMDAR1*, and *AtDHAR1* (Supplementary Figure S7). In contrast to drought stress response gene expression, oxidative stress response gene expression showed no significant variation between transgenic and control lines under normal conditions. Consistent with the *AtSOD1* expression pattern, SOD activity in transgenic and control lines showed no statistically significant variation under normal conditions. However, during drought stress (7 d), the SOD activity in transgenic lines was slightly reduced, while 5 days of drought exposure caused no change in SOD activity (Supplementary Figure S8). 

### 3.4. BrSR45a Overexpression Improved Basal Drought Tolerance in Seed Germination

**Figure 6:**
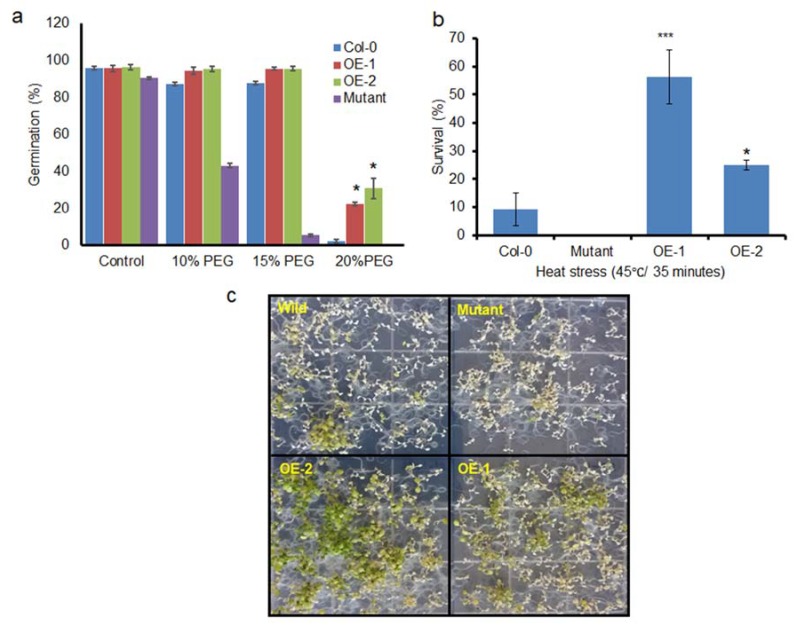
Assessment of stress tolerance efficiency of *BrSR45a* overexpressors, mutants, and control lines subjected to drought and heat stresses. The basal drought tolerance was assessed by using a seed germination assay at PEG6000-induced drought stress under greenhouse conditions (**a**) and the heat shock responses was tested by immersing the plants in a water bath at 45 °C for 35 min and then allowing them to recover for five days in a growth chamber (**b**,**c**). (** represents the statistical significance calculated from triplicate measurements).

Seed germination is one of the most critical stages for plant survival; hence, the basal stress tolerance efficiency of transgenic lines during seed germination was evaluated under PEG-induced drought stress conditions (Figure 6a). Sterilized seeds from OEs and Col-0 were sown on 3MM filter papers that were moistened with different percentages of PEG solution. The germination assays revealed that there was no increase in the variation of the germination rate in the presence of 10–15% PEG compared with that in the control; however, the germination rate of mutants at 15% and 20% and the germination rate of Col-0 at 20% PEG were reduced to a greater extent than in the transgenic lines. The OE lines showed a higher drought tolerance index (DTI; calculated as mentioned in Section 2.4) that ranged from 25–38%, while Col-0 and mutants showed a zero DTI during 20% PEG-induced drought stress. It is evident from the results that seed germination was constrained by PEG-induced drought stress. A high concentration (20%) of PEG almost completely inhibited the germination of the control, while 22.2% of the OE-1 line seeds and 34.38% of the OE-2 line seeds germinated, indicating that the overexpression of *BrSR45a* improved the basal drought tolerance level in transgenic plants in the presence of drought stress.

### 3.5. Basal therMotolerance of Transgenic Seedlings

The ability of seedlings to survive and remain photosynthetically active from heat shock stress can be considered as basal heat stress tolerance. As described previously [26], 9-d-old seedlings of OEs, mutants, and controls grown on plates were immersed in a water bath for 35 min at 45 °C. Heat stress tolerance was estimated as the percentage of surviving seedlings after a recovery period of 8 d at 23 °C (Figure 6b,c). The results showed that seedling viability was completely lost in *SR45a* loss of function mutants, while more than 50% of the OE-2 seedlings remain viable when subjected to heat stress. Similarly, around 20% of the OE-1 seedlings withstood heat stress and stayed with green leaves, the rate of survivability was higher in comparison with control seedlings. The heat phenotypes of OE-2 and OE-1 were consistent with *BrSR45a* expression pattern indicating *BrSR45a* possibly associated with heat stress responses. 

### 3.6. Disruption or Overexpression of SR45a Alters the Splicing Pattern of Genes Interacting with SR45a and Drought Stress Response Target Genes

**Figure 7:**
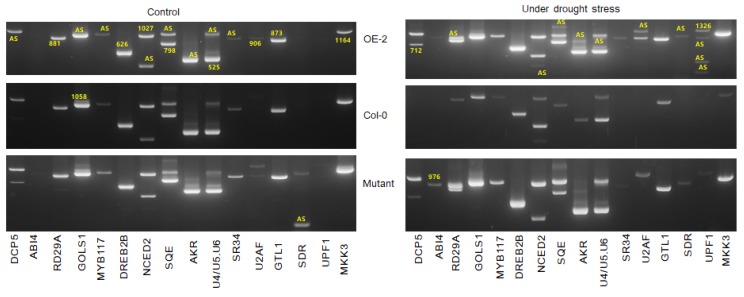
Differential alternative splicing of some of the SR45a-interacting genes and known drought-response genes in leaf samples collected from *BrSR45a* overexpressing, knock-out mutants (SALK_052345) and wild (Col-0) type Arabidopsis lines under control and drought stress (15 d) conditions. AS represents the alternatively spliced transcripts while, the numbers represents the length (in nucleotides) of the original transcripts.

The impact of the overexpression and loss of function of SR45a on the splicing patterns of drought tolerance genes were investigated (Figure 7). The splicing pattern of all the genes in OEs and Col-0 under normal conditions appeared almost similar, while the differential splicing of *DCP5, RD29A, GOLS1, AKR*, and *SDR* were observed between OEs and mutants. 

Moreover, the overexpression of SR45a did not alter the normal splicing of those genes unless the plants were exposed to drought stress conditions. Interestingly, the splicing patterns of most genes in the mutants under normal conditions were similar to those in the drought-stressed OE lines, suggesting that a loss of function would trigger stress response-induced alternative splicing. In general, the splicing patterns of *DCP5, RD29A, GOLS1, NCED2, SQE, AKR*, and *UPF1* in OEs and *NCED2, SQE, AKR,* and *UPF1* in mutants were different from those in Col-0, indicating that these genes could play a vital role in stress responses and alternative splicing when plants encountered stress signals from the environment. In comparison, the splicing patterns of *NCED2, AKR*, and *UPF1* were different between OEs and mutants, while all other tested genes showed a similar splicing pattern during drought stress. Two additional splice variants with slightly higher molecular sizes than the original amplicons were observed for *AKR* in the OE lines; however, the challenge is to uncover the precise functions of each splice variant, warranting the characterization of the splice variants. Some classes of AKRs were previously implicated in osmolyte production, which confers drought tolerance. Although no clear information is available for the mechanism by which the higher molecular weight splice variants of *AKR* were produced, the molecular weight of new splice variants were higher than the original transcripts. We found four variants of the UPF1 gene during drought stress in OE, and the size of the variant was much smaller than the expected product size, presumably indicating the presence of truncated transcripts. However, further characterization is needed to understand their functional roles in drought stress responses. Moreover, we revealed the functional linkage between SR45a and key post-transcriptional modifications via the NMD pathway through the alternative splicing of UPF1. In comparison with those in the respective controls, the splicing patterns of *DCP5, RD29A, NCED2, SQE, AKR*, and *UPF1* in OEs and *RD29A, GOLS1*, and *SDR* were different during drought conditions. Among the drought-tolerance genes, the splicing of *DCP5, SQE, AKR, SDR, GOLS1, NCED2, UPF1,* and *RD29A* were different under normal and drought stress conditions, suggesting that the alternative splicing of those genes is regulated by *SR45a.*

Apart from drought-responsive genes, *SR45a* also differentially alters the splicing of its interacting partners such as *U2 SnRNP auxiliary splicing factor small subunit* (Bra010926.1) and *U4/U6-U5 tri-snRNP-associated protein 2-like* (Bra013613.1) in OEs and mutants. In OEs, *U2AF35A* was alternatively spliced and produced 2 mRNA variants, possibly altering the splicing of other genes. The importance of splice variants during drought stress is unknown. Nonetheless, it is confirmed that the altered expression of *BrSR45a* controls the splicing pattern of *U2AF* in response to drought stress. Similarly, *Bra013613.1* produced three and two splice variants in OEs and mutants, respectively, during drought stress. These results suggest that *BrSR45a* alters the splicing of its interacting partners in addition to that of other target genes.

## 4. Discussion

Our previous study showed that abiotic stresses—including salt, drought, oxidative and heat shock—upregulate the expression of *BrSR45a* in *B. rapa* [15]. Herein, we characterized the functional role of *BrSR45a* in drought and heat stress responses. The promoter analysis showed that the SR45a promoter sequences had three important cis-acting elements that were responsive to abiotic stress. Further, the exogenous application of stress hormones, ABA, heat, and drought stress regulated *BrSR45a* expression (Figure 1). Previously, high-light irradiation and salinity stress were shown to increase the expression of *atSR45a* [13]. These results clearly indicate that *SR45a* play a critical role in abiotic stress responses of plants. The phenotyping of SR45a knock-out mutants with either control or *BrSR45a* overexpressors revealed no aberrant phenotypes for leaves and roots between them. This result corroborates the earlier findings [27], where the phenotypes of *AtSR45a* mutant lines were similar to that of wild types. However, difference in photosynthesis rate and degree of tolerance between mutants and overexpressors was observed during drought stress. The comparative analysis of the OJIPs showed that the O-J, J-I, and I-P phases in the overexpressors were positive and relatively higher than those in mutants indicating SR45a associated with photosynthesis efficiency under stress conditions. Maintaining a better photosynthesis rate under stress conditions expected to have positive impact on drought tolerance [27]. In fact, the first report on SR45a had demonstrated positive correlation between SR45a overexpression and photosynthesis efficiency in Arabidopsis [28].

Drought tolerance is an important agronomic trait contributed by a complex network of genetic factors. The role of ABA in drought tolerance is inevitable because of its critical role in modulating stress responsive gene expression to coordinate plant stress responses, growth, and development under adverse conditions. AtSR45a also participates in ABA signal transduction to modulate abiotic stress responses [5,22,29]. In this study, we found that expression of stress-related genes such as *AtDREB2A, AtNCED3, AtKIN1*, and *AtRD29A* were upregulated in transgenic lines compared with control plants, indicating that the overexpression of *BrSR45a* might induce stress response gene expression in transgenic plants. Previous studies on *DREB2A, NCED3*, and *RD29A* showed that their overexpression in *Robinia pseudoacacia*, grapevines and *A. thaliana,* respectively, were associated with improved tolerance to drought stress [30,31,32]. KIN1 produces LEA class proteins that aid in damage repair during stress [33]. Therefore, the induced expression of stress-related genes in SR45a overexpressors expected to alleviate the drought stress mediated negative impacts in plants [34]. Contrastingly, oxidative stress response gene expression (e.g., *APX1*, *SOD1*, *DHAR*, and *MDAR*) showed no significant variation between transgenic and control lines under normal conditions. Consistent with the *AtSOD1* expression pattern, SOD activity in transgenic and control lines showed no statistically significant variation under normal conditions suggesting that the expression of *SR45a* and oxidative stress are not correlated. Interestingly, *SR45a* homologs in Arabidopsis was unperturbed by reactive oxygen species [13] indicating possible shared functions for SR45a genes between these species. Furthermore, it is confirmed that BrSR45a do not alternatively spliced under hydrogen peroxide mediated oxidative stress while splice variants in *B. rapa* were reported [15]

Seed germination is one of the most critical stages for plant survival [25] and germination rate under drought stress indicates the degree of basal tolerance mechanism [35]. The overexpression of *BrSR45a* in transgenic lines had germination rate around 22–34% while the germination of mutants and control was completely inhibited in 15% or 20% PEG-induced drought stress. It is evident from the results that the overexpression of *BrSR45a* improved the basal drought tolerance level in transgenic plants in the presence of drought stress. Therefore, we investigated the association of *BrSR45a* with heat stress responses in this study by incubating 9-day-old seedlings of OEs, mutants, and controls grown on plates 45 °C (Figure 6c,d) for 35 min [26]. The viability of OE-2, OE-1, control and *SR45a* mutants were positively correlated with SR45a expression under stress conditions. Transgenic lines with highest expression of *BrSR45a* had better survivability while the loss of function causes severe susceptibility to heat stress. This result clearly indicates that *BrSR45a* overexpression resulted in heat-tolerant phenotypes in Arabidopsis seedlings. However, it is not clear *BrSR45a* or its splice isoforms induced during heat stress [15] contributing tolerance phenotypes, and characterization of heat stress derived splice variants will shed light on the precise role in thermotolerance.

Notably, evidence from the literature has shown that stress response genes in plants undergo alternative splicing [27]. SR45a proteins are important splicing factors in regulating gene expression through AS. The *SR45a*-dependent alternative splicing of target protein-encoding genes might have distinct biological functions [9,10] or may simply operate as part of the mRNA decay pathway for RNA surveillance system during unfavorable conditions [19]. To obtain knowledge on SR45a mediated alternative splicing of drought stress responsive genes both under normal and drought stress conditions, we investigated the splice variants of SR45a overexpressors, mutants, and controls. The comparative analysis of alternative splicing suggested that both enhanced activity and loss of activity change the splicing pattern in target genes but differentially. This suggests that SR45a is crucial for the splicing of target genes, including drought-responsive genes. Moreover, the overexpression of SR45a did not alter the normal splicing of those genes unless the plants were exposed to drought stress conditions. The splicing patterns of *NCED2, AKR*, and *UPF1* were different between OEs and mutants during drought stress exposure. AKRs are primarily involved in the detoxification of toxic aldehydes and ketones produced during stress [36]. NCED (9-cis-epoxycartoenoid dioxygenase), a key regulator of ABA biosynthesis and its overexpression, has been implicated in drought tolerance [31]. UPF1, a key NMD factor, activates the NMD pathway upon its phosphorylation [37]. However, the challenge is to uncover the precise functions of each splice variant, warranting the characterization of the splice variants. Nevertheless, some classes of AKRs were previously implicated in osmolyte production, which confers drought tolerance. The splice variants of *AKR* were slightly higher in molecular weight than the original amplicons in the OE lines. The possibility of intron retention being the prime reason for higher molecular weight splice variants can be ruled out on the basis of earlier findings that the high-light induced expression of *atSR45a* actively suppressed the efficiency of the intron-retention type mechanism [27]. In some cases, these transcripts can be degraded through the NMD pathway [38]. Despite that, SR45a-induced alternative splicing can be linked with stress tolerance in transgenic plants [39].

Apart from drought-responsive genes, *SR45a* also differentially alters the splicing of its interacting partners (*U2 SnRNP auxiliary splicing factor small subunit* (Bra010926.1) and *U4/U6-U5 tri-snRNP-associated protein 2-like* (Bra013613.1)) in OEs and mutants. A change in the expression of the U2 snRNP auxiliary splicing factor produced no variation in phenotypes [40] indicates other regulatory roles although the mechanism is not clear. Additionally, studies have documented that *SR45a* participates in the multiples roles, including glucose signaling pathway [18], the nonsense-mediated mRNA decay pathway (NMD) in the RNA surveillance system [19], ABA signaling [20], the autonomous flowering pathway [21], and stress response gene expression pathways. In this study, we showed that *BrSR45a* is involved in drought stress responses through the alternative splicing of drought stress response genes. In addition, we found that the overexpression of *BrSR45a* protects photosystem II during drought stress and improves basal thermotolerance in young seedlings. In conclusion, *SR45a* overexpression is positively correlated with enhanced drought tolerance, thermotolerance, and drought recovery rates compared with that observed in the *SR45a* mutant and Col-0 in an expression-dependent manner. Moreover, the OE and mutant lines differentially regulated several known drought-responsive genes, possibly contributing to enhanced drought tolerance. Although a precise understanding that supports SR45a-mediated drought tolerance is lacking, this study revealed that *BrSR45a* mediated the alternative splicing of target genes and their interacting partner genes to possibly contribute to stress tolerance in a concentration-dependent manner. By discovering the role of SR45a in drought and heat stress responses and the alternative splicing of target genes during stress responses, this work will be helpful for the development of stress-resilient crops to improve crop productivity under adverse environmental conditions.

## Supplementary Materials

The following are available online at https://www.mdpi.com/2073-4425/11/2/182/s1, Figure S1: BrSR45a promoter sequences with marked region of potential promoter motifs, Figure S2: PCR based genotyping for selection of homozygous *SR45a* mutants from SALK_052345 seedlings, Figure S3: Chlorophyll a fluorescence transients (OJIP curves) in dark adopted leaves of Arabidopsis seedlings *BrSR45a* overexpressors, Col-0, mutant-6 and mutant-8 subjected to drought stress (15 DAS), Figure S4: BrSR45a interaction with other proteins predicted by string-db online tools, Figure S5: Expression of stress markers under different time interval (0, 3rd, 5th, and 8th days) of progressive drought stress, Figure S6: *BrSR45a* expression pattern in selected transgenic lines (OE-2, OE-1), mutants (*SR45a* knock-out) and Col-0, Figure S7: The expression pattern of antioxidative enzyme genes in selected transgenic lines (OE-2, OE-1) and control (Col-0), Figure S8: Measurement of Superoxide dismutase (SOD) enzyme activity during different times of drought stress (d-0, 5, 7) in in selected transgenic lines (OE-2, OE-1), and control (Col-0) Arabidopsis plants, Table S1: List of primers and their annealing temperature used in this study.
