# Supplementary material for: Brassica Rapa SR45a Regulates Drought Tolerance via the Alternative Splicing of Target Genes
Source: Genes (Basel). 2020 Feb 10;11(2):182. doi: 10.3390/genes11020182 (PMC7074037; doi:10.3390/genes11020182)

**Supplementary Figure S1.** BrSR45a promoter sequences with marked region of potential promoter motifs

GACCAAGATAAAGTTTGCATCACAAAACGAGTTGGAGATGGGTATTATTATTATTATTACCAACCTTTTTGACTCCATAA
CTTGTGCCCGCTGTTCTGGGATCTTATCAGCTTCACTCGCTGTATAGCTAAAACATGATTCAATATAAAATATATTAACA
AAGCCTTAAACTTTTTATGCTTCACATTCCAAAAACCTTATTGTTGACAAACCTAGAGGGATGAGCAGAAGGAGGGAC
GAGAGGAGCATAAGCGGTTTCTCTTTCAGCAGCTGTCTGCTGAGCTTTCTGAAACTCTTTCAACACAGCTTGAAAGTC
CTTTGCAAGCTTAGCATCTGCAATCTTCTTACTTGGCTACAACAAACATTTAACAAACCATCATCATCAAACTCCAACAA
AACCAAAGAAGAAAAGATTGTGACTTTTACATTGACACCGCTTCGATGATCAGTTTCACTAGCTTCTTTAAGCTTAGCA
GAAGTATCCTTCACAAGCTGTCCTATATGCAACCTTGTCTTGTGCCTATAACGCAAATCAACCATTAGAGTCAATTCTG
AGCAGCTACAAAAAAATAAAATGATTTTTAAATTTCAATCTTACAGCTTCTCACGGAGCTCGGGCGTGTCTTTGGGAGT
ACCGAGCGTGTTAACGAGCCGCTGAAACGTGGAGACTCCGGTATTGATCTGGAAGATACCGGAAGCGACGGCTTGC
GTCGAATCTTGGCGACCACCACCACCGCCATTGATCTTCCTCGATCTTCCTTTCCCAGCTTCCAAATCTTGAAAACTCA
TCTTCTCTCTTTCGTGCCTATCTCTCTCTCTCTCCAACGATGAACGATTTGGTTTGAGAAGGGAAAATGATGAATGAGG
GGTCAAGGCGTGAATCGTGGAGACAGATAGACAGAACTTGAAAAGCGTTTTTTTTCTTA**TAACTG**ATTAGAAGAGATG
**MBS**
AAAGGCGAGATCAATTTTTGTTTCTTTCTGGTCCTTTCATTTCTATTTACTTACTAATGTCTCCTTATTTTATTGTTTAGT
GTAGGTTTGCATACTTTTGGCGTTTTAAGGTATAGAAAATTTAATAGTTTGTGTTTGATTTGGT**GTGCAT**TAGGAGTGG
**ABRE**
GACAGAAATCAACCTGAACTGAACCAAATTCTGACCAAACCAAAATATATATCCAAGAACATTTGATTGATATTTTTAAC
AACTTGAATAGTTTGTTTTTTTTTTGTTCGGTTTAAAACCGAACTGAACCAAAACCAATATATTTAGTAGTTAGCTTAAAT
AATATATTCATGATATTAAGATATTACCATCCTTTGATATAAACTCAACTTTTTAGCATTGATGATCAAAAGTGATGTTTG
TTTTCTTTTATGTTGTGTAGATTGCTTTTAAGTGAATTACACCAAATCCAAGCCAATATTAATTCGATATAAACCAAACTC
AACATAAATCAAACCGAATACAAATTAAACATAATCAAAATGGAATCAACCCAAAATTGAACCAAAATGGTCATGCCTA
ATTTATATACACAAAATCAGTTTCAGTAAACATTTAGTTGTAGTTTATTTTGGTTGTTCTCCATCTATTTAAAGTTACGGA
GCAGTCACAGTTTAACTATTCATAAATCGGCCGGGGGGTTAATTTATAATTAAGATATTCAGTGATGAGGTTACTTTAC
A**AGTGGCCGTCC**GACGTGGATGATTGTAATTGGTGGATAAAGTGATTATGTCACGTCTAAAATAATAAAGTATATGTTA
**CAT-Box CCGTCC-Box**
TTATTTAATTAAATGGAATCGTTCCCGACAAAGCAAAAGCTCTACGCACGACAACCTTCTCTGTCCCTTCACACGTATA
AAGTCGCCGGCGAGAGAAGAAGTTTCCGGACAGTCACGCGTGCCATTTTCTCAATATAAATAGAATTCACATTTTTGT
TTCTCACACCATC**AAAAATTTTC**TTTCTTCAAATTCGTTCTTCGCTGGTAAAAGAGAAAATCTTCCGTTACATCAGATTT
**HSE**
CTAAGAGTTTCTCAGCAATG

**Supplementary** Figure S**2.** PCR based genotyping for selection of homozygous SR45a mutants from SALK_052345 seedlings

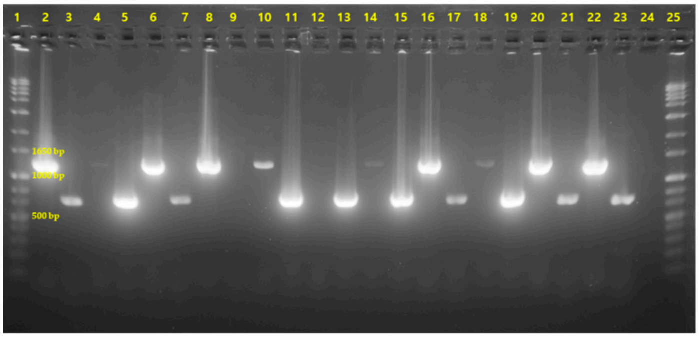


1-1 kb plus DNA ladder
2-W12, 3-T12: Heterozygous
4-W2, 5-T2: Homozygous
6-W3, 7-T3: Heterozygous
8-W4, 9-T4: Wild
10-W5, 11-T5: *
12-W6, 13-T6: Homozygous
14-W8, 15-T8: Homozygous
16-W9, 17-T9: Heterozygous
18-W10, 19-T10: Homozygous
20-W11, 21-T11: Heterozygous
22-W13, 23-T13: Heterozygous
24-Negative control
25-1 kb plus DNA ladder

Confirmatory analysis

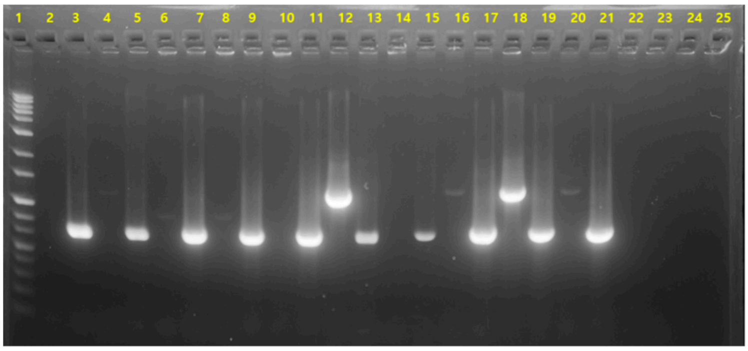


1-1 kb plus DNA ladder
2-W2, 3-T2 } Homozygous
4-W5, 5-T5 } Homozygous
6-W6, 7-T6 } Homozygous
8-W8, 9-T8 } Homozygous
10-W10, 11-T10 } Homozygous
12-W12, 13-T12 } **Heterozygous**
14-Negative control

To select homozygous mutant through PCR based genotyping (A two-step PCR genotyping assay) for further studies, the following primers were designed.

1. SR45F TCAAGCACTGAGAACAACGG
2. SR45R TGAACATGCTAAATCGAACCAC

Product size: 1239 bp

3. T-DNA LB ATTTTGCCGATTTCGGAAC

Product size: 602-902 bp

**Supplementary Figure** S**3.** Chlorophyll a fluorescence transients (OJIP curves) in dark adopted leaves of Arabidopsis seedlings BrSR45a overexpressors, Col-0, *SR45a* knock-out mutants (Knock-out mutant line 1 and 2 ) subjected to drought stress (15 DAS)

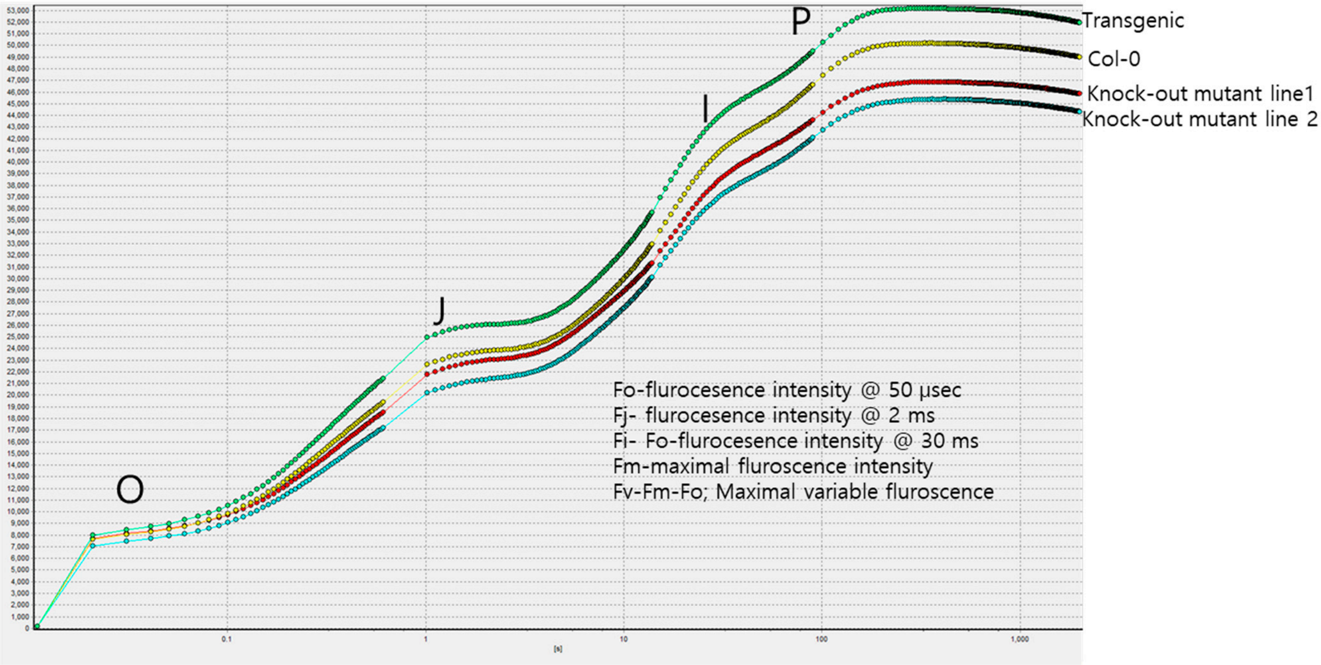

**Supplementary Table S1.** List of primers and their annealing temperature used in this study

| Primer Name | Sequence (5'->3') | Annealing Temp. | Product Size |
|---|---|---|---|
| **UPF1-FP** | ACTGTGGGTGTCGGAATGTC | 57°C/50 sec | 1326 bp |
| **UPF1-RP** | ACAACCGAAGATCAGCAGCA | | |
| **DCP5-FP** | ATGGCGGCTGATAATACGGG | 58°C/50 sec | 712 bp |
| **DCP5-RP** | ATGAAGAGGGTGCTTCTGGC | | |
| **ABI4-FP** | AATCCGATTCCACCACCGAC | 58°C/50 sec | 976 bp |
| **ABI-RP** | TCCAATGCTCTTGACCGACC | | |
| **DREB2B-FP** | GCAAAGTTCCTGCGAAAGGG | 57°C/50 sec | 626 bp |
| **DREB2B-RP** | AGGCCAACCGTAATCTGCAA | | |
| **NCED2-FP** | ATATCCGTAACGGCGCGAAT | 57°C/50 sec | 1027 bp |
| **NCED2-RP** | ATAGCCAAAAACGCGAACCG | | |
| **RD29A-FP** | CCACCAGGGACAAAGGTGTT | 58°C/50 sec | 881 |
| **RD29A-RP** | CCGTCAGATTCCGGCGAATA | | |
| **MKK3-FP** | GGAGCTATAGGAAGCGGAGC | 57°C/50 sec | 1164 |
| **MKK3-RP** | AAGCCCTTCTGCTTGGACTC | | |
| **SQE-FP** | AGCAGGAGAGGAACAAACCG | 57°C/50 sec | 798 |
| **SQE-RP** | CCATAAACCGCAACCGCAAA | | |
| **GTL1-FP** | TCAGGCTAACATTGCGGGTT | 59°C/50 sec | 873 |
| **GTL1-RP** | CTAGAACCACCGCCACTACC | | |
| **GOLS1-FP** | AGACAGCGATCGAGCTTACG | 58°C/50 sec | 1058 |
| **GOLS1-RP** | TATGTGTGGTTGTCGACCCG | | |
| **AKR-FP** | TACCTTGCGTCGGTCTAGGA | 57°C/50 sec | 727 |
| **AKR-RP** | TGAAGACCTTGTTGCTGCCA | | |
| **U4/U6.U5-FP** | TGAAAGCCAGCAAGAAACGC | 57°C/50 sec | 525 |
| **U4/U6.U5-RP** | ACACCTTCTCCCCTTCTGGT | | |
| **SR34-FP** | AGGCTTGCCTTCATCTGCTT | 57°C/50 sec | 907 |
| **SR34-RP** | AAGCAGACGTCTCCAGACAAG | | |
| **U2AF-FP** | ACTCGCGAACAAAGCAAACC | 57°C/50 sec | 906 |
| **U2AF-RP** | TTGAACCGTCCCTCTCTTGC | | |
| **PPI-FP** | CGCATGCGTCTGCATTCTAC | 56°C/50 sec | 1357 |
| **PPI-RP** | ACCCAATTGCAGAGAGTTGAAA | | |
| **MYB117-FP** | ACGGAAAAACAAGTGTGGATGG | 58°C/50 sec | 831 |
| **MYB117-RP** | TAGGCTACCTTGAAAGCAATGG | | |
| **SDR-FP** | ATGGCTTCCTCAGTCTCCTCT | 59°C/50 sec | 773 |
| **SDR-RP** | TTGCTGATTCTTGTGTCACCAC | | |

**Supplementary Figure S4.** BrSR45a interaction with other proteins predicted by string-db online tools

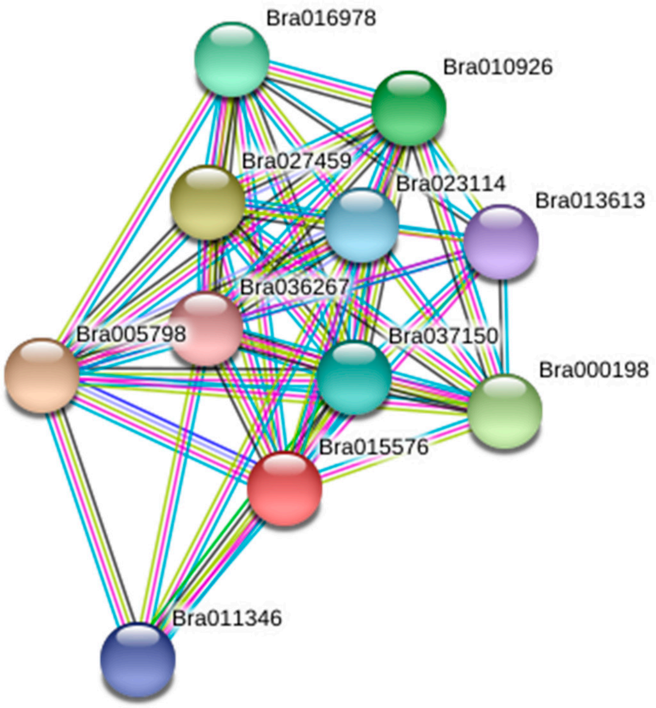


**Bra013613.1**=PREDICTED: U4/U6.U5 tri-snRNP-associated protein 2-like isoform X2 [Brassica rapa]

**Bra005798.1**-P=PREDICTED: glycine-rich RNA-binding protein RZ1C isoform X1

**Bra036267.1**-P=PREDICTED: serine/arginine-rich-splicing factor SR34 [Brassica rapa]

**Bra037150.1**-P=unnamed protein product [Brassica rapa],Sequence ID: VDC59016.1; 62% coverage with PREDICTED: ATP-dependent DNA helicase DDM1-like [Brassica rapa]

**Bra000198**.1-P=PREDICTED: pre-mRNA-splicing factor 38 [Brassica rapa]

**Bra015576**.1-P=PREDICTED: serine/arginine-rich splicing factor SR45a [Brassica rapa]

**Bra011346**.1-P=PREDICTED: peptidyl-prolyl cis-trans isomerase CYP95 isoform X1 [Brassica rapa]

**Bra016978**.1-P=Pre-mRNA splicing factor-38 like

**Bra010926.**1-P=splicing factor U2af small subunit

**Bra027459**.1-P=splicing factor U2af small subunit B

**Bra023114**.1-P=serine/arginine-rich splicing factor RS2Z33

**Supplementary Figure S5.** Expression of stress markers under different time interval (0, 3rd, 5th and 8$^{th}$ days) of progressive drought stress

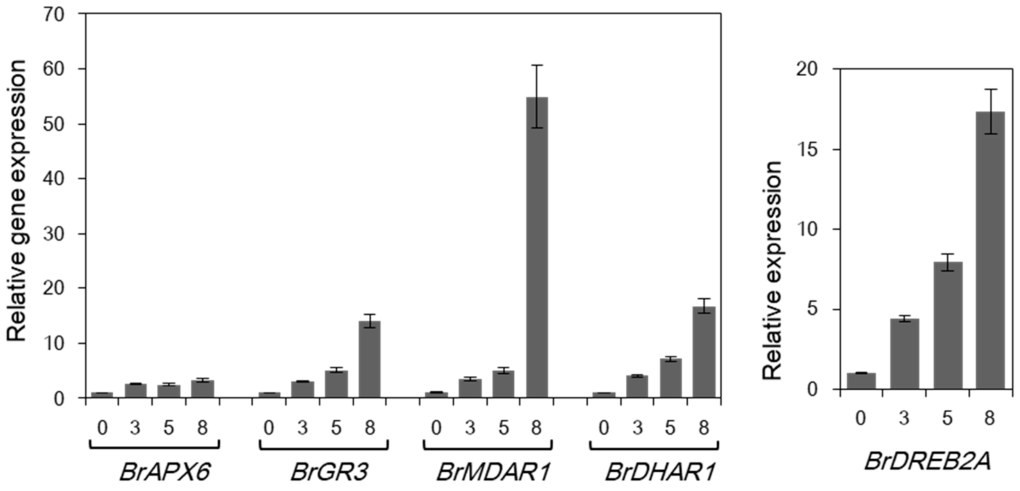


**Supplementary Figure S6.** *BrSR45a* expression pattern in selected transgenic lines (OE-2,OE-1), mutants (SR45a knock-out ) and Col-0.

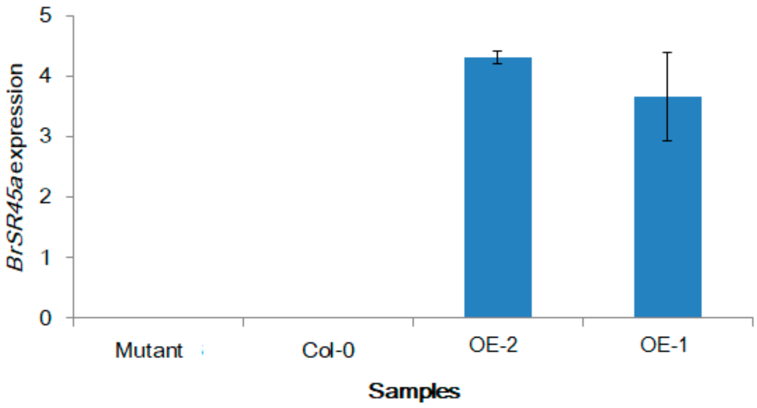

**Supplementary Figure S7. The expression pattern of antioxidative enzyme genes in** selected transgenic lines (OE-2,OE-1) and control (Col-0).

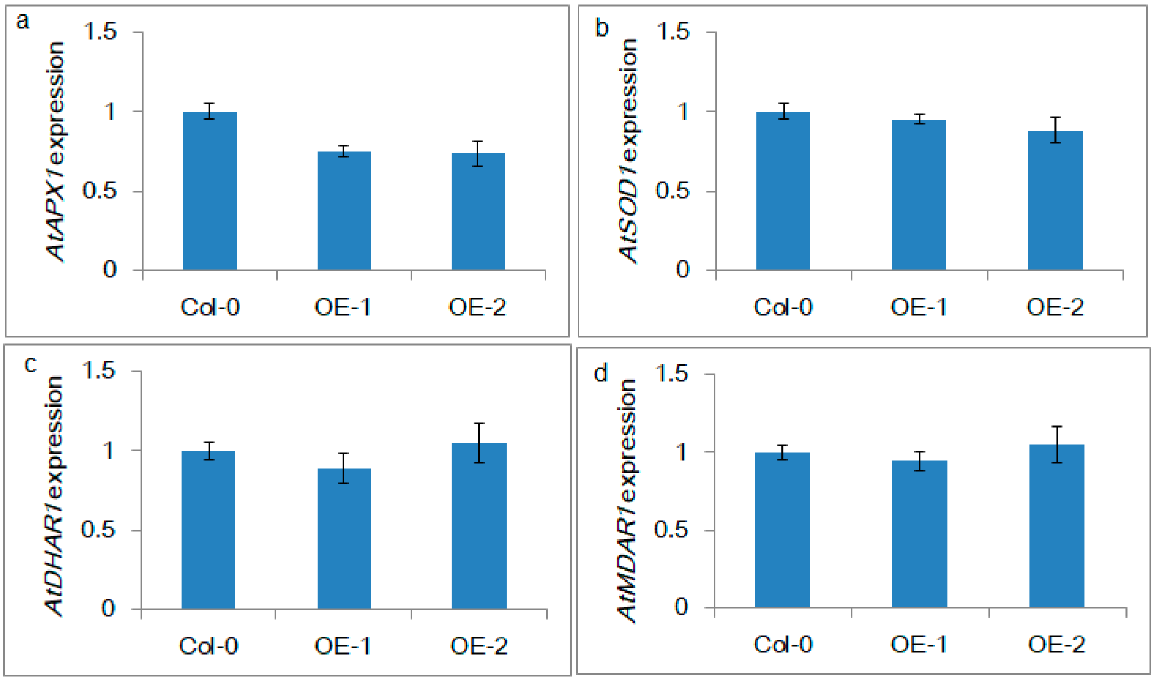

**Supplementary Figure S8**. Measurement of Superoxide dismutase (SOD) enzyme activity during different times of drought stress (d-0,5,7) in in selected transgenic lines (OE-2,OE-1), and control (Col-0) Arabidopsis plants.

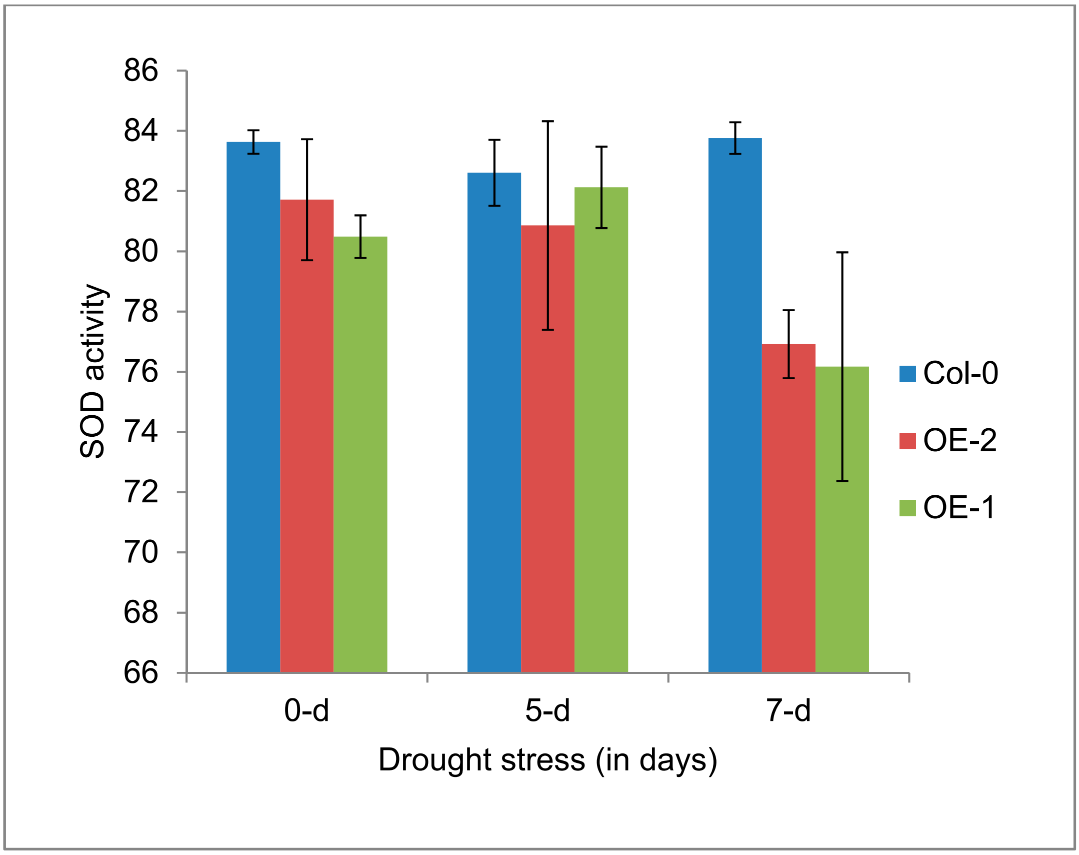

Supplement: Supplementary file 1 [file genes-11-00182-s001.pdf]
